# Self-Report Measures of Parental Self-Efficacy: A Systematic Review of the Current Literature

**DOI:** 10.1007/s10826-017-0830-5

**Published:** 2017-07-06

**Authors:** Anja Wittkowski, Charlotte Garrett, Rachel Calam, Daniel Weisberg

**Affiliations:** 1 School of Health Sciences, Division of Psychology and Mental Health, The University of Manchester, Manchester, UK; 2 Greater Manchester Mental Health NHS Foundation Trust, Manchester, UK; 3 Central Manchester NHS Foundation Trust, Manchester, UK

**Keywords:** Parental, Measure, Psychometric properties, Quality

## Abstract

Parenting self-efficacy (PSE) describes a parent’s belief in their ability to perform the parenting role successfully. Higher levels of PSE have consistently been shown to be correlated with a wide range of parenting and child outcomes. Consequently, many parenting interventions aim to improve PSE. PSE measurement has typically been via self-report measures. However, the wide range of available measures has resulted in their limited use, inconsistent terminology and ambiguous theoretical grounding. The purpose of this systematic review was to examine the psychometric and administrative qualities of the available PSE measures and offer clarity to the terminology and the theory underpinning their use so that the future use of PSE measures can be appropriate. Eleven electronic databases were searched. Articles were included if they introduced a new measure or were psychometric evaluations of an available measure of PSE for parents of children (from infancy until 18 years of age). Thirty-four measures were identified and their psychometric and administrative qualities were examined. Overall, the quality of the available measures was varied. Whilst this review makes recommendations regarding PSE measures for parents of infants through to adolescents, some caution should be applied when choosing the most appropriate measure. The theoretical grounding of each measure was clarified so that appropriate measures can be chosen under the relevant circumstances. The implications of refinement of the available measures are discussed and further research into improving PSE measurement is identified.

## Introduction

The term “self-efficacy” describes an individual’s belief in their ability to successfully perform a given task. Self-efficacy can inform how an individual may behave, indicating whether they attempt a task, how much effort they put into the task and how long they persist in the face of obstacles and aversive experiences (Bandura 1997, 2006). Bandura (1997) coined the term “self-efficacy” following the development of Social Cognitive Theory (SCT) (Bandura 1997), which offers an explanation for performance in certain tasks based on the reciprocity of a) personal factors (e.g., cognitive, biological and affective events), (b) environmental events and (c) behaviour (Crothers et al. 2008). According to Bandura and Adams (1977), individuals draw on four sources to gauge their self-efficacy: 1.Their interpretations of their own performance (e.g., successful performances are likely to raise self-efficacy, whereas less successful performances are likely to lower it). 2. Their own abilities by watching others perform a task. 3. Their response to social persuasion (e.g., encouragement or praise from others cultivates self-efficacy, whereas criticism reduces it) and 4. Their physiological and emotional states (e.g., confidence and happiness are more likely to instil a higher self-efficacy than anxiety and fear). These four sources were incorporated into a model of the relationship between self-efficacy and performance developed by Gist and Mitchell (1992), grounded in the SCT approach. They provided evidence that Bandura and Adams’ (1997) four sources of information in addition to three core processes determine self-efficacy. Firstly, there is an assessment of task requirements, which encourages reflections on the skills needed so that the task can be completed successfully. Second, there is an analysis of previous performances and attributions as to why the previous performance occurred in the way that it did. Thirdly, a detailed analysis of personal and situational factors takes place to assess the resources and constraints required in order to complete a task. Bandura’s (1988) work supported the notion that these processes are integrated with the four sources of information to form self-efficacy. The performance of the task is fed back into these sources to update the individual’s level of self-efficacy.

Parenting self-efficacy (PSE) can be defined as the caregiver’s or parent’s confidence about their ability to successfully raise children (Jones and Prinz 2005). However, parental or parenting self-efficacy (PSE) is often mislabelled as parental “confidence”, parental “competence” and parental “self-esteem” (Hess et al. 2004). In addition, these concepts are used inconsistently, with one concept being used when another would be more appropriate (e.g., Swick and Broadway 1997). Terminology has also been used interchangeably (e.g., MacPhee et al. 1996) or novel terminology has been introduced, such as “parental self-regulation” (Hamilton et al. 2014) and “parental self-agency” (Dumka et al. 1996).

Bandura (1997) argued that parental confidence refers to the strength of a belief about a task, but is not specific in what the strength of the belief is about, whereas PSE includes both strength of belief and an interpretation of capability based on that belief. Glidewell and Livert (1992) described parental confidence as stable over time; it is not situation-dependent or situation-specific. In contrast, they described PSE as situation-specific and variable according to the task and the context. Additionally, PSE is a theoretically defined construct, whereas confidence is a colloquial term unrelated to a specific theory (Pennell et al. 2012). Taking these ideas into account, De Montigny and Lacharité (2005) completed a conceptual analysis to demonstrate that parental confidence is indeed a separate concept to PSE. Similarly, they argued that parental self-esteem is a separate concept. Parental self-esteem is one’s judgement of worth as a parent, whereas PSE is one’s judgement of personal capability to fulfil the role of a parent (Bandura 1997). Parental competence is also a separate concept to PSE. It refers to the ability to complete a task successfully and efficiently (Pearsall and Hanks 1998), as does PSE, but it is based on others’ perspectives of how well the task will be completed, rather than a parent’s own judgement, as per PSE. The differences in concepts may be subtle, but they are important to consider because the correct terminology will ensure accuracy and consistency. An additional separate concept is parenting satisfaction: a subjective rating of contentment derived from being a parent, which influences PSE (Coleman and Karraker 2000; Rogers and White 1998). Thus, to remove all ambiguity, measures within this review specify which concept (PSE, confidence, esteem, competence or satisfaction) is investigated. We also included self-regulation because Hamilton et al. (2014) refer to the similarity between the above concepts and suggest that their combination results in “parenting self-regulation” which emphasises four distinct characteristics encompassing a general sense of parenting competence and confidence (self-efficacy, self-management, self-sufficiency and personal agency; Sanders 2000, 2008).

Clinical and research attention has been drawn to parenting self-efficacy, with two key reviews in this area to date (Coleman and Karraker 1998; Jones and Prinz 2005). Coleman and Karraker (1998) developed the meaning of the PSE construct, explored the relevant empirical findings and described the effect of PSE on parenting. Coleman and Karraker (1998) identified eight measures of PSE and provided some psychometric information on their reliability and validity. Their review was the first of its kind and evoked public and clinical interest. Jones **a**nd Prinz’s (2005) updated review provided further evidence that PSE is strongly correlated with positive parent and child psychological functioning, child adjustment, parenting competence and parenting satisfaction.

Both reviews offer consistent evidence that higher levels of PSE are strongly associated with an adaptive, stimulating and nurturing child-rearing environment, which encourages social, academic and psychological well-being. The evident importance of PSE has led to the development of interventions that target PSE so that the child-rearing environment can be improved. Interventions such as group-based parenting programmes that target parental empowerment have positively influenced PSE (see Wittkowski et al. 2016, for a detailed review), and positive change has been demonstrated to continue for at least a further 12 months (e.g., Guimond et al. 2008; Tucker et al. 1998).

PSE is usually assessed via self-report measures which is appropriate given that PSE reflects the parent’s belief in or judgement of his/her ability to successfully perform a given parenting task. Typically, measures assess the following four domains (e.g., Coleman and Karraker 2000): general or trait self-efficacy, domain-specific (also referred to as “task-related”), domain-general (also referred to as “global”) and narrow-domain (also referred to as “task-specific”). “General PSE” measures assess overall self-efficacy in the parenting role and items are not linked to specific parenting tasks (e.g., “What I do has little effect on my child’s behaviour.” Campis et al. 1986). Črn**č**ec et al. (2010) identified that these scales were suitable to a wide range of child ages, but are less sensitive to the tasks that face a parent of a child of a specific age. “Domain-specific” PSE measures assess parents’ beliefs in their ability to complete specific tasks of the parenting role for a child of a specific age (e.g., “How good are you at getting your baby to have fun with you?” Teti and Gelfand 1991). These measures offer greater sensitivity to specific tasks and ages, leading to greater predictive validity than general PSE measures (e.g., Marsh et al. 2002). Bandura (1997) argued that PSE is most accurate when assessed with domain-specific measures. ‘Domain general’ measures refer to functioning within one area of daily life, but do not specify the tasks or activities within which they must be performed (e.g., “I know good parenting tips that I can share with others.” Freiberg et al. 2014). Finally, “narrow-domain” focuses on one specific aspect of the parenting role, such as breastfeeding (Dennis and Faux 1999) or childbirth (Lowe 1993). The items are all task-specific, age-specific and situation-specific.

Despite this continued interest in PSE, to date there has been only one review of “parenting confidence” measures. In their review, Črn**č**ec et al. (2010) examined 28 measures of “parenting confidence”, which they used as an umbrella term to capture measures of self-efficacy (perceived by parents of infants to 12 year olds) that had been labelled in other ways (e.g., as sense of competence, self-definition or self-agency). They described each scale in detail, reported on several aspects of each scale’s reliability and validity, provided normative data where available, and ascribed an overall rating to each scale for the quality of its psychometrics based on a model used by Hammill et al.(1992).

In order to assist clinicians in assessing change in PSE and researchers in planning interventions, the current systematic review sought to update and extend current knowledge of PSE measures, completed by parents of children from birth to 18 years of age, by (a) providing detailed information on the psychometric and administrative properties of each identified measure, (b) stating the PSE domain being assessed, (c) clarifying terminology and (d) reviewing the theoretical grounding of each measure.

## Method

### Search Strategy

**Fig. 1:**
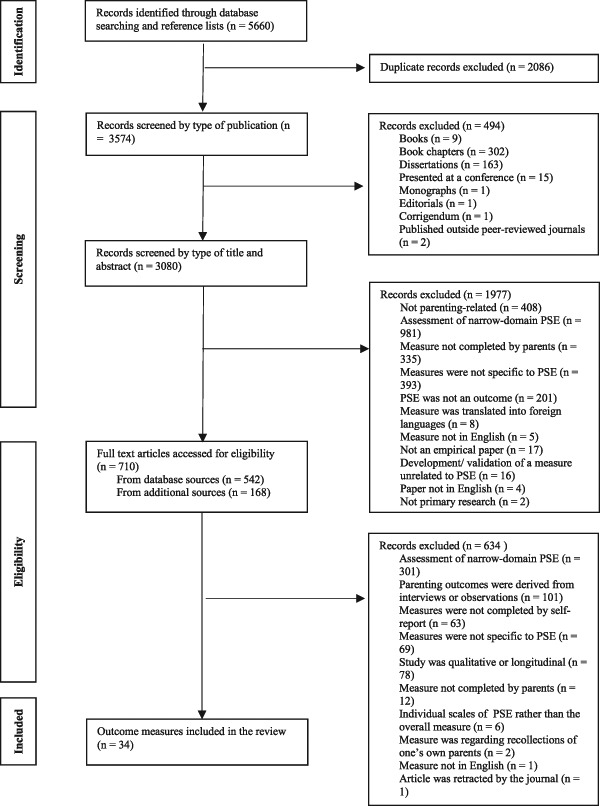
Schematic review of paper selection, based on the PRISMA guidance

A systematic search of ten online databases was conducted in December 2014 and updated in October 2016: OVID Maternity and Infant Care, Medline, PsycINFO, PsychARTICLES, EMBASE, Health and Psychosocial Instruments database, PubMed, Web of Science, CINAHL Plus and Google Scholar. The search strategy based on PRISMA guidance (2009) was developed to identify references relating to the development and psychometric properties of self-report measures of PSE. The earliest year of publication was restricted to 1970 to account for advances in PSE knowledge. The search terms were developed by combining terms specific to PSE measures. The search terms used, either in isolation or in combination, were: “Questionnaire*”, “outcome”, “measure*”, “parent* and (“self-efficacy” or “confidence” or “competence” or “esteem” or “satisfaction”) and “psychometric*.” The names of identified measures were used as terms for a further search of the above electronic databases. The reference lists from all identified papers were consulted alongside a review of measures (Črn**č**ec et al. 2010). Additionally, references of retrieved articles were screened for additional relevant studies. The search strategy and its results are described in Fig. 1.

### Inclusion and Exclusion Criteria

Self-report PSE measures were required to be developed in or translated to English with appropriate psychometric evaluation. Measures had to be applicable for parents of a child aged between the age of 0 (including pre-term infants) and 18 years. This age range was chosen to cover the span from infancy through to adolescence. Measures were included only if it was considered by the authors (on reviewing the scale content) that their primary focus was on self-efficacy but they could also assess other related constructs (competence, esteem, confidence, satisfaction, self-regulation). Broader measures that had a subscale of self-efficacy were not included unless the relevant subscale was validated independently (e.g., the Child Adjustment and Parenting Efficacy Scale; CAPES-SE, Morawska et al. 2014) or when the other subscale was also relevant to self-efficacy (e.g., the Parenting Sense Of Competence scale; PSOC, Johnston and Mash 1989, which has two subscales, labelled “satisfaction” and “self-efficacy”).

Measures were excluded if they did not investigate PSE and they were unpublished or had been published outside of peer-reviewed journals. Longitudinal and qualitative studies and those focussing on a “narrow-domain” were also excluded.

### Quality Assessment

Although several criteria for an evaluation of outcome measures exist (e.g., McDowell and Jenkinson 1996), some of the most comprehensive criteria have been proposed by Terwee et al. (2007), who drew on the Scientific Advisory Committee (SAC) of the Medical Outcomes Trust (SAC 2002) criteria. Terwee et al. defined eight attributes of measure properties that are essential to consider in a thorough high-standard evaluation: (1) content validity, (2) internal consistency, (3), criterion validity, (4) construct validity, (5) reproducibility, (6) responsiveness, (7) floor and ceiling effects and (8) interpretability. As part of the current review a further four criteria regarding the administrative properties of the measures and indicators of change based on Bot et al.’s (2004) ‘clinimetric checklist’ were added: (9) time to administer, (10) ease of scoring, (11) readability and comprehension and (12) minimal clinically important difference (MCID). These additional criteria offer practical information about the measures to which Terwee et al.’s checklist is not sensitive.

Consistent with Terwee et al.’s (2007) approach, each criterion was assigned a rating of “+” (clear description, above a specific threshold), “−” (clear description, below a specific threshold), “?” (description is lacking or is doubtful), or “0” (information is missing). The above ratings were coded so that a “+” achieved a score of 3, “−” achieved a score of 2, “?” achieved a score of 1 and “0” achieved a score of 0. Thus, each measure achieved a total score ranging between 0 and 36, with a higher score indicating stronger psychometric and administrative qualities. This score should only be used as a guide because it can incorrectly imply that all measurement properties are equally as important, yet readers should consider their choice of measure based on the presence of particular criteria. All measures were rated across the following domains:

#### Content validity (the extent to which the domain of interest is comprehensively sampled by the items in the questionnaire)

A clear description must be provided of the measurement aim, the target population, the concepts that are being measured and the process of item selection to obtain a score of 3. The target population must also be involved in item selection as well as experts. If there is no target population involvement in item selection but other criteria are met, then the measure scores 2. If a clear description of the afore mentioned aspects is lacking, if only the target population or experts are involved, or design and methods employed are doubtful, then the measure is awarded a score of 1. If no information is found on target population involvement, then the measure is awarded a score of 0.

#### Internal consistency (the extent to which items in a (sub)scale are inter-correlated, thus measuring the same construct)

A score of 3 is given when factor analyses (FA) have been performed on an adequate sample size (7* number of items and ≥100) AND Cronbach’s alpha(s) were calculated and fell between 0.70 and 0.95. If the criteria for FA are met and Cronbach’s alpha(s) calculated but they fall outside of the acceptable range (despite adequate design and method), then a score of 2 is given. If FA has not been performed or the study otherwise has doubtful design or method issues, this property scores 1. A score of 0 is awarded for no information on internal consistency.

#### Criterion validity (the extent to which scores on a particular questionnaire are related to a gold standard)

To obtain a score of 3, the authors must include convincing arguments that the gold standard against which the measure is being compared is “gold” AND the correlation with that gold standard must be at least 0.70. If the argument that the standard is gold is convincing but the correlation is less than 0,70 despite adequate design or method, the measure scores 2. If no convincing arguments are presented that the gold standard is “gold” or the design or method used to test the relationship is doubtful, the measure scores 1. If no information is found on criterion validity, the measure scores 0.

#### Construct validity (the extent to which scores on a particular questionnaire relate to other measures in a manner that is consistent with theoretically derived hypotheses concerning the measured concepts)

To score 3 on this property, specific hypotheses must be formulated AND at least 75% of the results must be in accordance with these hypotheses. If fewer than 75% of hypotheses made are confirmed, despite adequate design or method, then the measure scores 2. If the design or method of testing this property is doubtful (e.g., if no hypotheses are made with interpretations only made post-hoc) then the measure scores 1. If no information is found on construct validity then the measure scores 0.

#### Reproducibility: Agreement (the extent to which the scores on repeated measures are close to each other (absolute measurement error)

For a score of 3, reliability agreement should be assessed (test-retest or split-half) AND the authors should present one or more of the following: the limits of agreement (LOA), Kappa, Standard Error of Measurement (SEM), evidence that the minimal important change (MIC) is less than the smallest detectable change (SDC), or that the MIC is outside the LOA, or some other convincing argument that agreement is acceptable. If the MIC is greater than or equal to the SDC, or MIC equals or is inside the LOA, despite adequate design and method, the measure scores 2. If the design or method is doubtful, or MIC is not defined AND no convincing arguments that agreement is acceptable are made then, the measure scores 1. A score of 0 is given for no information on agreement.

#### Reproducibility: Reliability (the extent to which patients can be distinguished from each other, despite measurement errors [relative measurement error])

Authors must report the Intraclass Correlation Coefficient (ICC) or weighted Kappa value for the scale, which must be greater than or equal to 0.70, for a score of 3. Where the design and method are adequate but the ICC or weighted Kappa is less than 0.70, the measure scores 2. If the design and method by which this property has been assessed is doubtful, the measure scores 1. A score of 0 is given for no information on reliability.

#### Responsiveness (the ability to detect important change over time in the concept being measured)

To score 3 the authors must report the SDC, which must be less than the MIC, an MIC that is outside the LOA, or a RR that is greater or equal to 0.70. If the SDC is greater than or equal to the MIC, the MIC equals or is inside the LOA, the RR is less than or equal to 1.96 or the AUC is less than 0.70 despite adequate design and methods, then the measure scores 2. A score of 1 is given if the design or method used to test responsiveness is doubtful, while a score of 0 is awarded if no information on responsiveness presented.

#### Floor and ceiling effects (the number of respondents who achieved the lowest or highest possible score)

Less than or equal to 15% of respondents must have achieved the highest or lowest possible scores on the measure for a score of 3. If the number is greater than 15%, despite adequate design or methods, a score of 2 is awarded. If the design or method for ascertaining floor or ceiling effects is doubtful, the measure scores 1. If there is no information included on floor or ceiling effects, then the measure scores 0.

#### Interpretability (the degree to which one can assign qualitative meaning to quantitative scores)

To score 3, at least two of the following have to be reported: Mean and standard deviation scores of multiple groups, comparative data on distribution of scores, information on the relationship of scores to other measures or clinical diagnoses, or define MIC. A score of 2 is not assigned for this property. If the design or method of the part of the study designed to generate information about interpretability is doubtful, if fewer than two of the above are offered or MIC is not defined, a score of 1 is assigned. A score of 0 is given for no information on interpretation.

#### Time to administer (time needed to complete the measure, see)

Bot et al. 2004 For a score of 3 it was necessary to demonstrate that participants were able to complete the measure in less than or equal to 10 min. If they took longer than 10 min, the measure scored 2, while a score of 1 was given if the methods used to test the administration time were doubtful. If no information was included on administration time, the measure scored 1.

#### Ease of scoring (the extent to which the measure can be scored by a trained investigator or expert)

The total score for the scale had to be generated by summing items, the measure needed to use a visual analogue scale or the formula used to compute total score had to be a simple one (e.g., reversal of specific items) for a score of 3. Measures scored 2 if they used a visual analogue scale in combination with a formula or a complex formula on its own. If the method for combining items to generate an overall score was unclear, then the scale scored 1. If there was no information about scoring, then the measure obtained a score of 0.

#### Readability and comprehension (the extent to which the measure is understandable for all patients)

For a maximum score of 3 authors had to have tested readability using at least one of: (a) the Flesch Kinaid Reading Ease; (b) Flesch Kincaid Grade Level; (c) Gunning Fog Score; (d) Coleman Liau Index, or (e) Automated Readability Index. If readability was tested using at least one of these methods, but the result was inadequate, a score of 2 was given, If the method of assessing readability/comprehension was considered inadequate, the measure was awarded a score of 1. A score of 0 was given for no information on readability.

#### Minimally clinically important difference (MCID) (the smallest difference in score in the domain of interest which patients perceive as beneficial and would mandate a change in a patient’s management)

Measures were awarded a score of 3 if they presented a MCID. A score of 2 was not assigned in the case of this property. If a doubtful design or method had been used to calculate the MCID then the measure was assigned a score of 1, and if no information was presented, the measure scored 0.

### Inter-Rater Reliability

One member of the research team (DW) reviewed the psychometric properties of each measure and another researcher, independent to the research team, reviewed eight of the 34 measures (24%). The inter-rater correlation coefficient was found to be .91.

### Examination of Domains and Theoretical Grounding

As previously described, terms related to PSE (self-efficacy, satisfaction, competence, confidence) have not been used consistently in the literature. In order to provide clarification as to the construct being measured, revised constructs were assigned to each measure by the review authors. Secondly, based on an assessment of the scale content, the authors assigned each PSE measure to one or more of the domains identified by Coleman and Karraker (2000). The content of each measure was then analysed according to Gist and Mitchell’s (1992) overarching theoretical model of self-efficacy and the different components each covered were identified.

## Results

The database searches identified 5660 publications. Following the strict application of the inclusion and exclusion criteria, a total of 76 studies referring to 34 self-report PSE measures were included in this review (see Fig. 1).

### Child’s Age

**Fig. 2:**
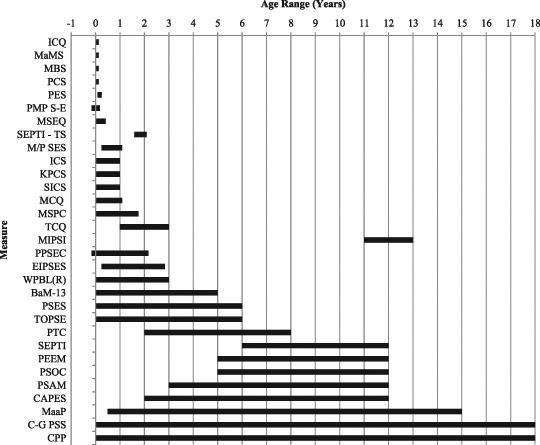
Age ranges of children for each measure. *Note*: The BAP and KPSS have been omitted because their age ranges were not identified. Measures are ordered by length of the age range, from shortest to longest

The majority of the measures were for parents of infants (preterm-13 months) and toddlers (14–36 months) (*n* = 17) (see Fig. 2). One measure was designed for toddlers and pre-schoolers (the Fathering Self-Efficacy Scale, FSES; Sevigny et al. 2016). There was no measure specifically for parents of pre-schoolers (3–5 years), only one measure for school-age children (5–12 years) (e.g., the Parent Empowerment and Efficacy Measure, PEEM; Freiberg et al. 2014) and no measures specifically for adolescents (13–18 years). Many measures were instead developed for a range of ages. Three measures, the Me as a Parent (MaaP, Hamilton et al. 2014), the Cleminshaw-Guidubaldi Parenting Satisfaction Scale (C-G PSS, Guidubaldi and Cleminshaw 1989) and the Comfort with Parenting Performance (CPP, Ballenski and Cook 1982), covered the widest range of ages.

### Number of Items and Sub-scales

The number of items ranged from three to 82 (*m* = 26.74, SD = 18.15). The KPSS had the fewest (3 items), whereas the Tool to Measure Parenting Self-Efficacy (TOPSE, Kendall and Bloomfield 2005) had the most (82 items). Many measures had just one subscale (*n* = 16) but other measures included multiple subscales (*n* = 18), ranging from two (e.g., BAP) to nine (TOPSE). The number of subscales on two measures, the Maternal Self-Confidence Paired Comparisons (MSPC, Seashore et al. 1973) and the Self-Efficacy for Parenting Tasks Indexes (SEPTI-TS, Van Rijen et al. 2014), was unclear.

### Content Validity

Eighteen of the measures (52.94%) received the highest rating of 3 for content validity (indicating that the measurement aim, target population, concepts being measured and process of item selection were clearly described by the authors, and that the target population were involved in item selection as well as experts). One measure (2.94%) scored 2, indicating that there was no target population involvement in item selection, but that other criteria had been met Ten measures (29.41%) scored 1, indicating that a clear description of the aforementioned aspects was lacking, that only the target population or experts, not both, were involved, or that the design and methods used to ensure content validity were doubtful. Five measures (14.71%) scored 0, indicating that no information could be found on target population involvement. These five measures were all included in articles in which the primary aim was experimental and required the use of a measure. In contrast, the 18 measures with the highest possible score of 3 were in articles in which the primary aim was an investigation into the psychometric properties of a measure.

### Floor and Ceiling Effects

Only ten of the measures (29.41%) offered information on floor and ceiling effects. Of these, eight measures (23.53%) obtained the maximum score of 3. One measure scored 2 points, indicating that sufficient information had been presented, while one measure suggested the presence of floor and ceiling effects, the authors did not offer sufficient information to determine their presence.

### Internal Consistency

Fourteen of the 34 measures (41.18%) achieved the maximum score of 3 for this property, indicating that factor analyses had been performed on the scale with an adequate sample size (7* the number of items and ≥100), that Cronbach’s alpha had been calculated for each subscale identified, and that these value fell between 0.70–0.95. Twelve measures did not complete a factor analysis or their methods were ambiguous resulting in a score of 2, whereas seven measures (20.59%) offered no information about internal consistency. The Infant Care Questionnaire (ICQ, Secco 2002) obtained a score of 1 because the authors reported internal consistency statistics, but these were inadequate.

### Criterion Validity

Only one measure, the What Being the Parent of a Baby is Like (Revised) (WPBL[R], Pridham and Chang 1989)-WPBL(R)), obtained the maximum score for this property for providing convincing arguments that there was a “gold standard” and their measure correlated well with this standard. As the C-G PSS and Parental Self-Agency Measure (PSAM, Dumka et al. 1996) referred to a gold standard, but the authors did not offer convincing arguments of their standard being “gold” (*n* = 2), these two measures obtained a score of 1. All other measures did not provide this information.

### Construct Validity

Thirteen of the measures (38.24%) achieved the maximum score for this property, indicating that the authors had formed specific hypotheses about the relationship between scores on their measure and other measures of theoretically related constructs, with 75% of their findings being in accordance with their hypotheses. Many of the remaining measures did not offer a clear assessment (*n* = 12) or any information on construct validity (*n* = 9).

### Agreement

Many authors offered information about how comparable scores were on the same measure on separate occasions, using a specified reliability agreement assessment (*n* = 16, 47.06%), with 16 measures obtaining the maximum score. Four measures obtained a score of 2 (11.76%) because the authors offered information on agreement but the result was inadequate. Three measures (8.82%) hinted at acceptable levels of agreement but did not offer sufficient information for which they scored 1. The remaining 11 measures (32.35%) did not refer to agreement or absolute measurement error.

### Reliability

With the exception of one measure (e.g., the Infant Care Questionnaire, ICQ; Secco 2002), no information was available on how parents could be distinguished from each other. The information provided by the ICQ suggested that the reliability was inadequate (meriting only a score of 1).

### Responsiveness

For four measures only, the authors reported on the responsiveness properties of their measures (11.76%). These included the Being a Mother scale (BaM-13, Matthey 2011), the Karitane Parenting Confidence Scale (KPCS, Ĉrnčec et al. 2008), the Self-Efficacy for Parenting Tasks Indexes – Toddler Scale (SEPTI-TS, Van Rijen et al. 2014) and the Parenting Sense of Competence Scale (PSOC, Johnston and Mash 1989). The authors of the paper concerning the PSOC referred to responsiveness, but did not offer sufficient information to warrant a rating higher than 1.

### Interpretability

Twelve measures achieved a rating of 3, offering details of how one can assign qualitative meaning to scores (35.29%). For 12 measures, authors reported some information on the scores obtained by their samples, but did offer adequate information, instead scoring 1 out of 3 (32.26%). The remaining measures (*n* = 11, 32.26%) did not offer any information on interpretability (and so were scored 0).

### MCID

Most authors did not report the MCID for their measures (*n* = 32, 94.12%). Only two measures (5.88%) (BaM-13 and KPCS) offered this information, obtaining the maximum score.

### Ease of Scoring

Most measures utilised a Likert scale from which responses were either summed or the mean score was calculated (*n* = 22, 64.71%). The ICQ utilised a visual analogue scale and the Myself as a Mother and My Baby Scale (MaMS and MBS, Walker et al. 1986) utilised a semantic differential scale. However, these measures were scored by similar methods and therefore all of these measures score obtained the maximum score. Two measures, the Perceived Competence Scale (PCS, Rutledge and Pridham 1987), which obtained a score of 3, and the Parenting Self-Efficacy Scale (PSES, Purssell and While 2013), which obtained a score of 0, utilised both Likert scaled items and dichotomous items. Nine measures (26.47%) did not offer information on how to obtain a score.

### Time to Administer

Although Bot et al. (2004) suggested that measures taking longer than 10 min to complete were less desirable than measures that took less time, their choice of time limit was arbitrary and has only been reproduced for consistency. Six measures (17.64%) obtained a maximum score for administration times of under 10 min, whereas two measures (5.88%) (MaaP and TOPSE) had reported administration time of over 10 min and hence obtained a score of 2. Two measures, the KPCS and the Maternal Confidence Questionnaire (MCQ, Zahr 1991), included some but insufficient information to determine administration time, resulting in a score of 1. The authors of the remaining measures (*n* = 23, 67.65%) did not include any information about administration time.

### Readability and Comprehension

Only four measures included reliability and comprehension information. Information for the Child Adjustment and Parent Efficacy Scale (CAPES) and C-G PSS suggested that readability and comprehension levels were adequate (these measures scored 3), whereas the MCQ and PCS referred to readability and comprehension but did not offer sufficient detail to warrant a score of more than 1.

### Overall Quality

All scores are presented in Table 1 but should be used as a guide only. No measure achieved a perfect score of 36 and the scores varied from 1 to 28 (*m* = 12.67, median = 14.00, SD = 6.52). The KPCS obtained the highest score of 28, while the MSPC obtained the lowest score of 1. Table 2 provides a description of each PSE measure.

**Table 1:** Ratings achieved by each measure following the summary of the quality assessment

Measure	Psychometric properties										Administrative properties			Overall score (0–36)
	Content validity	Floor and ceiling effects	Internal consistency	Criterion validity	Construct validity	Reproducibility		Responsiveness	Interpretability	MCID	Ease of scoring	Time to administer	Readability and comprehension	
						Agreement	Reliability							
APT	2	2	3	0	1	0	0	0	0	0	3	0	0	11
BaM-13	3	0	3	0	3	2	0	3	1	3	3	3	0	24
BAP	0	0	1	0	1	0	0	0	0	0	0	0	0	2
CAPES-SE	3	0	3	0	1	3	0	0	3	0	3	0	3	19
C-G PSS	3	0	1	1	3	3	0	0	0	0	0	0	3	14
CPP	1	0	0	0	0	3	0	0	1	0	0	0	0	5
EIPSES	3	0	1	0	3	3	0	0	3	0	3	0	0	16
FSES	3	3	2	0	3	3	0	0	0	0	3	0	0	17
ICQ	3	0	1	0	3	3	1	0	1	0	3	0	0	15
ICS	3	0	1	0	1	3	0	0	0	0	3	3	0	14
KPCS	3	3	3	0	3	3	0	3	3	3	3	1	0	28
KPSS	1	1	0	0	1	3	0	0	3	0	0	0	0	9
MaMS	0	0	3	0	0	2	0	0	3	0	0	0	0	8
MaaP	3	0	3	0	0	3	0	0	1	0	3	2	0	15
MBS	0	0	3	0	0	2	0	0	3	0	0	0	0	8
MCQ	1	0	3	0	1	3	0	0	1	0	3	1	1	14
MSEQ	1	0	1	0	0	0	0	0	0	0	3	0	0	5
M/P SES	1	0	3	0	1	0	0	0	0	0	3	0	0	8
MIPSI	0	0	0	0	0	0	0	0	1	0	3	0	0	4
MSPC	0	0	0	0	0	0	0	0	1	0	0	0	0	1
PCS	1	0	1	0	1	0	0	0	0	0	3	0	1	7
PEEM	3	3	3	0	3	3	0	0	1	0	0	0	0	16
PES	3	0	1	0	1	0	0	0	0	0	3	3	0	11
PMP S-E	3	3	3	0	3	3	0	0	3	0	3	3	0	24
PPSEC	3	0	1	0	3	1	0	0	1	0	3	0	0	12
PSAM	3	0	3	1	3	1	0	0	1	0	3	3	0	18
PSES	1	3	0	0	0	0	0	0	0	0	0	0	0	4
PSOC	1	0	3	0	1	2	0	1	3	0	3	0	0	14
PTC	1	0	0	0	0	0	0	0	3	0	3	0	0	7
SEPTI	1	3	3	0	3	0	0	0	3	0	3	0	0	16
SEPTI – TS	3	3	1	0	3	3	0	3	3	0	3	0	0	22
TCQ	3	0	1	0	1	3	0	0	0	0	3	3	0	14
TOPSE	3	0	0	0	1	3	0	0	3	0	3	2	0	15
WPBL(*R*)	3	3	1	3	3	1	0	0	1	0	3	0	0	18

*APT* assessment of parenting tool (Moran et al. 2016), *BaM-13* being a mother (Matthey 2011), *BAP* being a parent (McMahon et al. 1997), *CAPES–SE* child adjustment and parent efficacy scale (Morawska et al. 2014), *C–G PSS* Cleminshaw–Guidubaldi parenting satisfaction scale (Guidubaldi and Cleminshaw 1989), *CPP* comfort with parenting performance (Ballenski and Cook 1982), *EIPSES* early intervention parenting self-efficacy scale (Guimond et al. 2008), *FSES* fathering self-efficacy scale (Sevigny et al. 2016), *ICQ* infant care questionnaire (Secco 2002); *ICS* infant care survey (Froman and Owen 1989), *KPCS* Karitane parenting confidence scale (Črn**č**ec et al. 2008), *KPSS* Kansas parental satisfaction scale (James et al. 1985), *MaMS* and *MBS* myself as a mother scale and my baby scale (Walker et al. 1986), *MaaP* me as a parent (Hamilton et al. 2014), *MCQ* maternal confidence questionnaire (Zahr 1991), *MSEQ* maternal self-efficacy questionnaire (Fish et al. 1991), *M/P SES* maternal / paternal self-efficacy scale (Teti and Gelfand 1991), *MIPSI* multicultural inventory of parenting self-efficacy (Dumka et al. 2002), *MSPC* maternal self-confidence paired comparisons (Seashore et al. 1973), *PCS* perceived competence scale (Rutledge and Pridham 1987), *PEEM* parent empowerment and efficacy measure (Freiberg et al. 2014), *PES* parent expectation survey (Reece 1992), *PMP S-E* perceived maternal parenting self-efficacy (Barnes and Adamson‐Macedo 2007), *PPSEC* preterm parenting self-efficacy checklist (Pennell et al. 2012), *PSAM* parental self-agency measure (Dumka et al. 1996), *PSES* parenting self-efficacy scale (Purssell and While 2013), *PSOC* parenting sense of competence scale (Johnston and Mash 1989); Ohan et al. 2000), *PTC* parenting tasks checklist (Sanders and Woolley 2005), *SEPTI* self-efficacy for parenting tasks indexes (Coleman and Karraker 2000), *SEPTI-TS* self-efficacy for parenting tasks indexes—toddler scale (Van Rijen et al. 2014), *TCQ* toddler care questionnaire (Gross and Rocissano 1988), *TOPSE* tool to measure parenting self-efficacy (Kendall and Bloomfield 2005), *WPBL(R)* what being the parent of a baby is like (revised) (Pridham and Chang 1989)

**Table 2:** Description and additional information of the PSE measures

Measure	Target population	Study population(s)	Constructs cited by authors	Revised Constructs	Number of scales	Number of items	Number of response options	Range of Scores	Components of Gist and Mitchell (1992) model assessed	Domain
APT	0 to 24 months	1376 American parents (89% female)	Efficacy	Efficacy	2	30–37	5 (to 5)	30–150 (30 item)	1, 2c, 3, 4	Domain-specific and domain-general subscales
BaM-13	0 to 5 years	630 Australian mothers	Satisfaction	Efficacy; Esteem	3	13	4 (0 to 3)	0–39	2c, 3	Domain-specific
BAP	0	Australian	Competence; satisfaction	Efficacy; Satisfaction	2	46	0	0	3	General
CAPES-SE	2 to 12 years	347 Australian parents	Efficacy	Efficacy	1	20	10 (1 to 10)	20–200	1, 2b, 3, 5	Domain-general
C-G PSS	0 to 18 years	130 parents	Satisfaction	Efficacy; Confidence; Satisfaction	5	50	4	0	2b, 2c, 3, 5	General
CPP	0 to 18 years	278 mothers	Competence	Efficacy	1	8 to 14	6 (1 to 6)	8–48 to 14–84	2a, 2b, 2c, 3, 5	Domain-specific
EIPSES	3 to 34 months with disability	112 USA mothers	Competence; Efficacy	Efficacy	1	20	7 (1 to 7)	7–140	3	Domain-specific
FSES	Preschool children	224 Canadian fathers; 247 Canadian fathers	Efficacy	Efficacy	1	20	9 (1 to 9)	20–180	1, 2c, 3, 4, 5	Domain-specific
ICQ	0 to 6 weeks	264 Canadian mothers	Competence	Efficacy	3	38	6 (visual analogue) (0 to 5)	1–5	2b; 3	Domain-specific
ICS	0 to 12 months	142 parents	Efficacy	Efficacy	1	51	5 (A to E)	0	2b, 2c, 3	Domain-General
KPCS	0 to 12 months	187 mothers	Efficacy	Competence; Efficacy	3	15	4 (0 to 3)	0–45	2a, 2c, 3	Domain-specific
KPSS	0	1980: 84 USA mothers 1984: 85 USA mothers, 52 USA fathers	Satisfaction	Efficacy; Satisfaction	1	3	5	0	3, 4, 5	Domain-general
MaMS	1 day to 6 weeks	122 mothers	Confidence	Efficacy	1	11	7 (semantic differential scale)	0	2b, 2c, 3, 4	Domain-general
MaaP	6 months to 15 years	300 Australian parents	Regulation	Efficacy	4	16	5 (1 to5)	16–80	2a, 3, 4, 5	Domain-general
MBS	1 day to 6 weeks	122 mothers	Confidence	Efficacy	1	6	7 (Semantic differential scale)	0	2b, 2c, 3, 4	Domain-general
MCQ	0 to 13 months	43 mothers	Confidence	Efficacy	1	14	5 (1 to 5)	70	1, 3	Domain-specific
MSEQ	0 to 5 months	83 USA mothers	Competence; Efficacy	Efficacy	3	9	6	0	2a, 2b, 2c, 3	Domain-specific
M/P SES	3 to 13 months	86 mothers (48 diagnosed with depression)	Competence; Efficacy	Competence; Efficacy	1	10	4	0	1, 2a, 2b, 2c, 3, 5	Domain-specific
MIPSI	11 to 13 years	161 two-parent USA families	Competence; Efficacy	Efficacy	1	17	5	0	2b, 2c, 3	Domain-specific
MSPC	Birth to 21 months	32 mothers of premature infants	Confidence	Efficacy	2	30	2	0–100 (% responses ‘mother’ vs. other caregiver)	2c, 3	Domain-specific
PCS	0 to 6 weeks	140 mothers	Competence	Efficacy	1	68	6 (1 to 6). Some closed questions.	0	3	Domain-specific
PEEM	5 to 12 years	866 Australian parents	Confidence	Efficacy	1	20	10 (1 to 10)	20 to 200	2a, 2b, 2c, 3	Domain-general
PES	1 to 3 months	105 first-time mothers	Efficacy	Efficacy	1	20	11 (0 to 10)	0 to 20	1, 3, 4, 5, 6	Domain-specific
PMP S-E	Preterm to 1 month	165 UK mothers	Efficacy	Efficacy	4	20	4 (1 to 4)	20 to 80	1, 2c	Domain-specific
PPSEC	Preterm to 24 months	155 Australian parents	Competence; Efficacy	Efficacy	3	36	7 (1 to 7)	36 to 252	1,2b, 3	Domain-specific
PSAM	3 to 12 years	94 Spanish speaking & 90 English speaking mothers, SW USA	Agency*	Efficacy	1	10 (5 item available)	7 (1 to 7)	10 to 70	1,3	Domain-general
PSES	0 to 6 years	152 UK parents of child during ill health	Efficacy	Efficacy	4	18	Likert 4; dichotomous	0	3	Domain-specific
PSOC	5 to 12 years	220 mothers and fathers	Competence; Efficacy; Esteem; Satisfaction	Efficacy; Satisfaction	2	17	6 (1 to 6)	17 to 102	2a, 2b, 2c, 3, 6	Domain-general
PTC	2 to 8 years	124 Australian mothers	Efficacy	Efficacy	2	14/ subscale	100 (0 to 100)	0–100 (mean) separate for each subscale	3, 4	Domain-specific
SEPTI	6 to 12 years	145 mothers	Efficacy	Efficacy	5	36	6 (1 to 6)	1 to 6	1,3	Domain-specific
SEPTI—TS	19 to 25 months	68 mothers from E USA	Efficacy	Efficacy	7	53	6 (1 to 6)	53 to 318	3,4,5	Domain-specific
TCQ	1 to 3 years	49 mothers	Confidence	Efficacy	1	36	5 (A to E)	0	2a, 2c, 3	Domain-general
TOPSE	0 to 6 years	82 UK parents	Efficacy	Competence; Efficacy	9	82	11 (0 to 10)	0	2a, 2b, 2c, 3	Domain-specific
WPBL(R)	0 to 3 months	93 mothers	Satisfaction; Efficacy	Satisfaction; Efficacy	3	25	9 (1 to 9)	25 to 225	2b, 2c, 3	Domain-specific

*Note* A score of “0” indicates that the information is missing

### Measure Domain

Each measure was ascribed one or more domains according to the Coleman and Karraker (2000) model. No narrow-domain measures were selected because these had been excluded from the review on screening. Twenty-one measures assessed only domain-specific PSE, while ten measures assessed only domain-general PSE. One measure, the Assessment of Parenting Tool (APT, Moran et al. 2016) assessed both domain-specific and domain-general self-efficacy, and two measures, the C-G PSS and the BAP, assessed general self-efficacy.

### Terminology

Constructs were assigned to each measure. According to measure authors, 19 of the measures assessed PSE and 12 of those assessed PSE only. Following the strict application of the construct definitions described, it was found that the majority of measures investigated only PSE (*n* = 25), whereas the remainder investigated a combination of constructs (*n* = 9).

### Theoretical Grounding

**Fig. 3:**
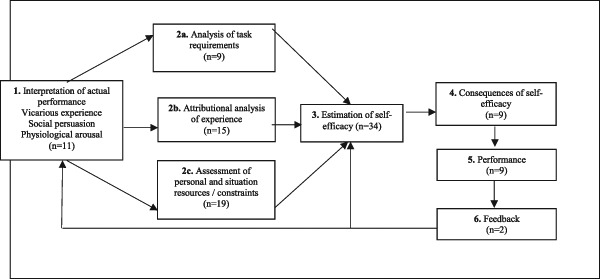
The frequency of measures that were retrospectively described by Gist and Mitchell’s (1992) theoretical approach to self-efficacy. *Note*:Adapted from “Self-Efficacy: A Theoretical Analysis of its Determinants and Malleability” by M. E. Gist, and T. R. Mitchell, 1992, *Academy of Management Review, 17*(2). Copyright 1992 by Academy of Management Publications. Permission to reprint not required

Although all measures were developed for a specific need (e.g., FSES for fathers), some authors did not discuss any relationship to the available PSE literature or its theoretical approaches (e.g., the Being a Parent [BAP], McMahon et al. 1997; and the Kansas Parental Satisfaction Scale [KPSS], James et al. 1985). For this review, all 34 measures were ascribed a theoretical grounding based on Gist and Mitchell’s (1992) model of self-efficacy (see Fig. 3). Although all included estimations of self-efficacy, only four of the measures exclusively assessed estimations of self-efficacy. Many of the measures also identified part of the assessment prior to forming PSE: Analysis of task requirements (*n* = 9), attributional analysis of experience (*n* = 15) and/or assessment of personal and situation resources/constraints (*n* = 19). Several measures were grounded in Bandura’s (1982) hierarchy of influence that forms PSE (*n* = 10). In contrast, based on Gist and Mitchell’s (1992) model relatively few measures investigated the consequences of PSE (*n* = 9), the performance based on the estimated PSE (*n* = 9) and the feedback of the performance (*n* = 2) (see Fig. 3).

## Discussion

PSE has been demonstrated to be a strong predictor of parenting functioning and is an important target for intervention. There are a large number of measures of PSE and related concepts currently available often developed for particular research studies. However, this can create problems in comparing and integrating knowledge concerning PSE, hampering progress in our understanding of how PSE is formed, operates and how it can be modified. Inconsistent use of terminology, the variety of theoretical models used to inform scale development and problems with reliability and validity can also contribute to inconsistencies in the literature. Administrative properties (e.g., number of items, ease of scoring, etc.) of available measures also vary, some measures being more suitable for certain types of research or clinical context than others. Our aim with this review was to provide up-to-date information for clinicians and researchers to help guide their choice of measures by systematically reviewing the literature of available measures, clarifying terminology and assessing the quality of identified measures in terms of their psychometric and administrative properties. In addition, we sought to enhance comparability of measures and theoretical clarity by situating each measure within a single, overarching, evidence-based model of self-efficacy (Gist and Mitchell 1992).

The current review builds on the findings of a previous review of measures of “parenting confidence”. Črn**č**ec et al. 2010 initially identified 36 measures but excluded five, meaning that they eventually included 31 measures in their review. They counted one of these measures (a single measure that has four versions) as four separate measures, whereas we counted this as a single measure. Thus, Črn**č**ec et al. (2010) included 28 unique measures. Although the current as well as the 2010 review examined psychometric properties of measures, the aim of each review differed which is reflected in the modest overlap in the measures included by Črn**č**ec et al. (2010) and ourselves. Focusing on more strictly defined PSE measures for parents of children up to 18 years of age, we included only 18 of the 28 measures mentioned by Črn**č**ec et al. (2010) alongside 16 additional measures. A number of these measures were measures published after 2010, which also means that our review provided updated information. In contrast to Črn**č**ec et al. (2010) who provided a summary rating for a measure based on currently available data on its psychometric properties, we used the quality rating tool by Terwee et al. (2007). This tool assesses the psychometric quality of the initial development and validation work carried out on each scale. The Terwee et al. (2007) checklist appraises more psychometric properties and is therefore more comprehensive. In the current review, the ratings in each of the areas contributing to the overall rating were made more transparent with the aim of guiding the reader to measures strong in the particular areas of validity or reliability that are important to them in their research or clinical work.

This review highlights that some measures have undergone rigorous psychometric evaluations, as evidenced by a maximum score. The psychometric properties mostly rigorously examined include content validity (18/34 obtained a maximum score), agreement (16/34), internal consistency (14/34), construct validity (13/34) and interpretability (12/34). In contrast, far less attention has been paid to the assessment of reliability, MCID, responsiveness and criterion validity. The paucity of information in those areas may reflect the lack of a “gold standard” or the opportunity for extensive psychometric evaluation as part of one study. It was noticeable that of the 18 measures demonstrating excellent content validity, all 18 were reported in studies of psychometric properties.

The 34 measures varied in the reported quality of their psychometric and administrative properties: the KPCS was the only measure scoring in the top quarter. Twelve measures obtained scores placing them in the lowest quarter, with the MSPC obtaining the lowest score. Although the total quality rating score should only be seen as a guide, researchers and clinicians could consider the following measures: the Perceived Maternal Parenting Self-Efficacy (24/36) (PMP-SE, Barnes and Adamson‐Macedo 2007) for parents of pre-term infants; KPCS (28/36) (Črn**č**ec et al. 2008) for parents of infants (0 to 12 months), the SEPTI-TS (22/36) (van Rijen et al. 2014) for parents of toddlers (13 to 36 months); BaM (24/36) for parents of pre-school-aged children (3 to 5 years); CAPES-SE (19/36) for the parents of school-aged children (5 to 12 years) and the MaaP (15/36) (Hamilton et al. (2014) for parents of adolescents (12 years+). The latter is a general PSE measure, and is unlikely to be sensitive to the issues pertinent to parents of an adolescent child. On a related note these measures are a selection of domain-general and domain-specific measures, underpinned by different theoretical backgrounds.

Twenty-one of the 34 measures were domain-specific measures which assess parents’ beliefs in their ability to complete specific tasks. According to Črn**č**ec et al. (2010), these measures are more sensitive to the tasks undertaken by parents of a child with a specific age. Due to their specificity, Marsh et al. (2002) argued that these measures have greater predictive validity than general measures of PSE. Incidentally, only two general PSE measures were included, one of which covered the widest age range possible (0–18) and the other did not specify any age range. Given these facts, clinicians and researchers are advised to choose a measure guided by their research or clinical needs and to consider if a domain-specific PSE measure is appropriately applicable across multiple developmental stages (like the Comfort with Parenting Performance [CPP], Ballenski and Cook 1982).

The current review found some evidence that the terminology used within the literature is inconsistent. Following a concept analysis (De Montigny and Lacharité 2005) the terminology was clarified and the subtle difference between concepts was clarified. The results identified that the terms “efficacy”, “esteem”, “competence” and “confidence” seemed to be used interchangeably. Some authors clarified their use of terminology. For example, Črn**č**ec et al. (2010) referred to measures of PSE but explained that they preferred the term “confidence” to ease the reader’s understanding. Whilst this rationale was clear, it unintentionally re-introduced ambiguity into this area of research. In addition, incorrect terminology was noted in measures that included an incorrect concept within their title. For example, the title “Maternal Confidence Questionnaire” (MCQ; Zahr 1991) informs the reader that confidence is under investigation, whereas PSE would have been more appropriate. Similarly, the “Parental Self-Agency Measure” (PSAM, Dumka et al. (1996) utilised an entirely new label for PSE. Although it is unlikely that incorrect terminology will cause any confusion, the terminology is inappropriate from a purely theoretical standpoint.

All PSE measures within the review fitted the Gist and Mitchell’s (1992) model as a theoretical framework for process of self-efficacy. Only a few measures included items that were at the very start of this theoretical process, based on Bandura and Adams’ (1997) four sources of information that form self-efficacy. This suggests that the majority of available measures work on the assumption that parents have already attempted a task at hand, and perceptions of PSE have already been developed. Further evidence is seen in the relatively small number of measures included within the first of Gist and Mitchell’s (1992) three assessments following the initial formation of self-efficacy (analysis of task requirements). Gist and Mitchell (1992) suggest that an analysis of task requirements is only necessary when the task is novel or has not been attempted. If a task has been performed before, the individual is likely to rely on their interpretation of previous performance (attributional analysis of experience). As there were more measures within this type of assessment, it is clear that the majority of measures in this review tend to investigate PSE *after* it has been initially formed.

Parents who attend structured parenting courses may be encouraged to develop their existing skills. However, parents may also be taught new skills. For professionals who wish to measure PSE for new tasks, this is most accurate when using measures that can be described by Bandura and Adams’ (1997) sources of information and Gist and Mitchell’s (1992) analysis of task requirements (e.g., CAPES, KPCS or MaaP).

The second of the three assessments is the attributional analysis involved in PSE judgements. This analysis involves an individual’s attributions as to why a particular performance level occurred. Although an attributional analysis is necessary to estimate PSE, it is insufficient without an examination of a third assessment, the availability of specific resources and constraints so that a task can be performed. This assessment accounts for personal factors, such as skill level, anxiety and desire, and situational factors, such as competing demands and distraction. The result of this assessment is likely to determine future performance. Gist and Mitchell (1992) argued that any measure of one, two or all of these assessment processes result in information that helps to identify levels of PSE. Therefore, every measure that is grounded in at least one of three assessments also offers an estimation of PSE. Users of measures are encouraged to consider this causal link when interpreting their results.

Only a small number of measures were identified that regard the processes after an estimation of PSE is made, suggesting that measures within these theoretical areas have obtained less attention than the processes that help to determine PSE. Paradoxically, there is a great deal of consistent research on the consequences of PSE (as summarised in Coleman and Karraker 2000), indicating that greater levels of PSE have beneficial and therapeutic consequences for individuals. This incongruity may be understood if one considers that there is no clinical or therapeutic need for additional measures within these areas because the benefits of higher PSE are already documented. This can be demonstrated in parenting interventions (e.g., Sanders and Woolley 2005) which offer measurements of the change in PSE during the intervention (e.g., educating parents on how to better interact with their children), rather than measuring changes to the consequences of increased PSE (e.g., parenting levels of stress or improvements in the quality of parent-child interactions). This latter measurement may not be necessary.

Our review prioritised accurate terminology, theoretical grounding in SCT and the administrative and psychometric properties of the available measures but some limitations have to be considered. Although Terwee et al.’s (2007) and Bot et al.’s (2004) criteria provided a framework for a thorough evaluation, the subjective nature of identifying “gold standards” and the seemingly arbitrary use of time-limits and specific thresholds between “adequate” and ”inadequate” have to be acknowledged. These criteria were chosen for their comprehensiveness and a measure that performs well against these criteria is likely to be an appropriate and robust choice, but as previously stated any total scores need to be viewed as a guide for selection only. There are other ways of identifying the strengths of a measure as highlighted by Črn**č**ec et al. (2010) review of all available data on a measure and their examination of particular aspects, such as the provision of normative data.

With regards to the theoretical underpinnings of a measure, it is possible that measure authors simply stated they were referring to a particular theory without providing any further empirical support. By reviewing the content, each measure was ascribed a theoretical grounding based on Gist and Mitchell’s (1992) model of self-efficacy, in an attempt to clarify the measure’s theoretical grounding.

The current review demonstrates that adequate PSE-specific measures with good psychometric and administrative properties exist. Whilst the current review includes measures suitable for mothers and fathers (e.g., MaaP) and mothers alone (e.g., BaM-13), in 2010 Črnčec, Barnett, and Matthey commented on the absence of measures specifically for fathers and so far only one measure for fathers has been developed (e.g., FSES for fathers). The use of more appropriate measures, sensitive to gender differences, strengthen research findings around paternal PSE (e.g., Hudson et al. 2003) and facilitate research into a better understanding of the difference in PSE between mothers and fathers.

Consideration should also be given to the construct of PSE, which is often viewed as either high or low. This dichotomous view has led to the possibly unhelpful comparisons of parents with higher PSE to parents with lower PSE. However, less is known about the parents who fall within the moderate range. This may be due to many measures including items that were linked to performance on specific tasks, which encourages an all-or-none estimation of efficacy. A further possibility, proposed by Coleman and Karraker (1997), is that individuals with moderate levels of self-efficacy do not perform as predictably on measures as individuals with more extreme scores. Perhaps further investigation and interpretation is necessary into measures that are sensitive to moderate scores of PSE. Similarly, as over 10 years have passed since Jones and Prinz’s review of the PSE literature, an updated review of empirical studies is warranted.

The current review identified that since 1970, 34 measures of PSE have been developed yet none have been widely adopted, indicating that measures might have been developed for specific applications. As PSE has been demonstrated to be a strong predictor of parenting functioning, its measurement should not be overlooked or assigned a minimal degree of importance in theoretical models of parenting or child development. Reliable, valid and efficient measurement of PSE permits individuals to document change in the parenting role and the resulting improvements to quality of life. Measures can ensure that parents with lower levels of PSE are better identified and supported to improve their skills in parenting. Consequently, they can be encouraged to develop the skills in which they feel unprepared. Once parents have conviction and belief in their own abilities, the quality of parenting can be optimised and the role of being a parent can become as pleasurable as possible. This systematic review enables users of PSE measures to identify the most appropriate theoretical and logistical measure for their needs.

## Electronic supplementary material

Supplementary Table S1

Supplementary Table S2

Supplementary Table S3

Supplementary Table S4

## References

[CR1] Ardelt M, Eccles JS (2001). Effects of mothers’ parental efficacy beliefs and promotive parenting strategies on inner-city youth. Journal of Family issues.

[CR2] Ballenski, C. B., & Cook, A. S. (1982). Mothers’ perceptions of their competence in managing selected parenting tasks. *Family Relations*, 489–494. http://www.jstor.org/stable/583923.

[CR3] Bandura A (1997). Self-efficacy: The exercise of control.

[CR4] Bandura A (2006). Toward a psychology of human agency. Perspectives on Psychological Science.

[CR69] Bandura, A. (1988). Organizational applications of social cognitive theory. *Australian Journal of Management*, *13*(2), 275–302.

[CR74] Bandura, A. (1982). Self-efficacy mechanism in human agency. *American Psychologist*, *37*(2), 122–147. doi:10.1037/0003-066X.37.2.122.

[CR68] Bandura, A., & Adams, N. E. (1977). Analysis of self-efficacy theory of behavioral change. *Cognitive Therapy and Research*, *1*(4), 287–310.

[CR5] Barnes CR, Adamson‐Macedo EN (2007). Perceived Maternal Parenting Self‐Efficacy (PMP S‐E) tool: development and validation with mothers of hospitalized preterm neonates. Journal of Advanced Nursing.

[CR6] Boivin M, Pérusse D, Dionne G, Saysset V, Zoccolillo M, Tarabulsy GM (2005). The genetic‐environmental etiology of parents’ perceptions and self‐assessed behaviours toward their 5‐month‐old infants in a large twin and singleton sample. Journal of Child Psychology and Psychiatry.

[CR7] Bot S, Terwee C, Van der Windt D, Bouter L, Dekker J, De Vet H (2004). Clinimetric evaluation of shoulder disability questionnaires: A systematic review of the literature. Annals of the Rheumatic Diseases.

[CR8] Broussard ER, Hartner MSS (1970). Maternal perception of the neonate as related to development. Child Psychiatry and Human Development.

[CR9] Bugental, D. B., & Cortez, V. L. (1988). Physiological reactivity to responsive and unresponsive children as moderated by perceived control. *Child Development*, 686–693. http://www.jstor.org/stable/1130568 3383677

[CR10] Campis LK, Lyman RD, Prentice-Dunn S (1986). The parental locus of control scale: Development and validation. Journal of Clinical Child Psychology.

[CR75] Coleman, P. K., & Karraker, K. H. (1997). Self-efficacy and parenting: Findings and future applications. *Developmental Review*, *18*, 47–85. doi:10.1006/drev.1997.0.

[CR11] Coleman PK, Karraker KH (1998). Self-efficacy and parenting quality: Findings and future applications. Developmental Review.

[CR12] Coleman PK, Karraker KH (2000). Parenting self‐efficacy among mothers of school‐age children: Conceptualization, measurement, and correlates. Family Relations.

[CR13] Coleman PK, Karraker KH (2003). Maternal self‐efficacy beliefs, competence in parenting, and toddlers’ behavior and developmental status. Infant Mental Health Journal.

[CR14] Črnčec R, Barnett B, Matthey S (2008). Development of an instrument to assess perceived self‐efficacy in the parents of infants. Research in Nursing and Health.

[CR15] Črnčec R, Barnett B, Matthey S (2010). Review of scales of parenting confidence. Journal of Nursing Measurement.

[CR67] Crothers, L. M., Hughes, T. L., & Morine, K. A. (2008). Theory and cases in school-based consultation: A resource for school psychologists, school counselors, special educators, and other mental health professionals. New York: Routledge Taylor & Francis Group.

[CR16] De Montigny F, Lacharité C (2005). Perceived parental efficacy: Concept analysis. Journal of Advanced Nursing.

[CR17] Dennis C-L, Faux S (1999). Development and psychometric testing of the Breastfeeding Self-Efficacy Scale. Research in Nursing and Health.

[CR18] Dumka, L. E., Prost, J., & Barrera Jr, M. (2002). The parental relationship and adolescent conduct problems in Mexican American and European American families. *Journal of Couple and Relationship Therapy*, *1*(4), 37–57. doi:10.1300/J398v01n04_02

[CR19] Dumka, L. E., Stoerzinger, H. D., Jackson, K. M., & Roosa, M. W. (1996). Examination of the cross-cultural and cross-language equivalence of the parenting self-agency measure. *Family Relations*, 216–222. http://www.jstor.org/stable/585293

[CR20] Elder Jr, G. H., Eccles, J. S., Ardelt, M., & Lord, S. (1995). Inner-city parents under economic pressure: Perspectives on the strategies of parenting. *Journal of Marriage and the Family*, 771–784. http://www.jstor.org/stable/353931.

[CR21] Fagot BI (1995). Development of a pleasure in parenting scale. Early Development and Parenting.

[CR22] Fish M, Stifter CA, Belsky J (1991). Conditions of continuity and discontinuity in infant negative emotionality: Newborn to five months. Child Development.

[CR23] Freiberg K, Homel R, Branch S (2014). The parent empowerment and efficacy measure (PEEM): A tool for strengthening the accountability and effectiveness of family support services. Australian Social Work.

[CR24] Froman RD, Owen SV (1989). Infant care self-efficacy. Research and Theory for Nursing Practice.

[CR25] Gibaud-Wallston, J., & Wandersmann, L. P. (1978). *Development and Utility of the Parenting Sense of Competence Scale*. Paper presented at the American Psychological Association, Toronto.

[CR70] Glidewell, J. C., & Livert, D. E. (1992). Confidence in the practice of clinical psychology. *Professional Psychology: Research and Practice*, *32* (5), 362–368.

[CR26] Gist ME, Mitchell TR (1992). Self-efficacy: A theoretical analysis of its determinants and malleability. Academy of Management Review.

[CR100] Gross, D., & Rocissano, L. (1988). Maternal confidence in toddlerhood: Its measurement for clinical practice and research. *The Nurse Practitioner*, *13*(3), 19–29. 3374866

[CR27] Guidubaldi J, Cleminshaw HK (1989). Development and validation of the Cleminshaw-Guidubaldi Parent-Satisfaction Scale. Journal of Clinical Child Psychology.

[CR28] Guimond AB, Wilcox MJ, Lamorey SG (2008). The early intervention parenting self-Efficacy Scale (EIPSES) scale construction and initial psychometric evidence. Journal of Early Intervention.

[CR29] Hamilton, V. E., Matthews, J. M., & Crawford, S. B. (2014). Development and Preliminary Validation of a Parenting Self-Regulation Scale: “Me as a Parent.” *Journal of Child and Family Studies*, *24*(10), 1–12. doi:10.1007/s10826-014-0089-z.

[CR30] Hammill, D. D., Brown, L. & Bryant, B. R. (1992). *A consumer’s guide to tests in print* (2nd ed.). Austin, TX: Pro-ed. Cited in Črnčec, R., Barnett, B., & Matthey, S. (2010). Review of scales of parenting confidence. *Journal of Nursing Measurement*, *18*(3), 210-240. doi:10.1891/1061-3749.18.3.210 10.1891/1061-3749.18.3.210 21290926

[CR31] Hess CR, Teti DM, Hussey-Gardner B (2004). Self-efficacy and parenting of high-risk infants: The moderating role of parent knowledge of infant development. Journal of Applied Developmental Psychology.

[CR32] Hudson DB, Campbell-Grossman C, Ofe Fleck M, Elek SM, Shipman A (2003). Effects of the new fathers network on first-time fathers’ parenting self-efficacy and parenting satisfaction during the transition to parenthood. Issues in Comprehensive Pediatric Nursing.

[CR33] James DE, Schumm WR, Kennedy CE, Grigsby CC, Selectman K, Nichols CW (1985). Characteristics of the kansas parental satisfaction scale among two samples of married parents. Psychological Reports.

[CR34] Johnston C, Mash EJ (1989). A measure of parenting satisfaction and efficacy. Journal of Clinical Child Psychology.

[CR35] Jones TL, Prinz RJ (2005). Potential roles of parental self-efficacy in parent and child adjustment: A review. Clinical Psychology Review.

[CR36] Kendall S, Bloomfield L (2005). Developing and validating a tool to measure parenting self‐efficacy. Journal of Advanced Nursing.

[CR37] Lowe NK (1993). Maternal confidence for labor: Development of the childbirth self‐efficacy inventory. Research in Nursing and Health.

[CR38] MacPhee D, Fritz J, Miller‐Heyl J (1996). Ethnic variations in personal social networks and parenting. Child Development.

[CR73] Marsh, H. W., Ellis, L. A., & Craven, R. G. (2002). How do preschool children feel about themselves? unraveling measurement and multidimensional self-concept structure. *Developmental Psychology*, *38*, 376–393. doi:10.1037/0012-1649.38.3.376. 10.1037//0012-1649.38.3.376 12005381

[CR39] Matthey S (2011). Assessing the experience of motherhood: the being a mother scale (BaM-13). Journal of Affective Disorders.

[CR40] McDowell I, Jenkinson C (1996). Development standards for health measures. Journal of Health Services Research.

[CR41] McMahon CA, Ungerer JA, Tennant C, Saunders D (1997). Psychosocial adjustment and the quality of the mother-child relationship at four months postpartum after conception by in vitro fertilization. Fertility and Sterility.

[CR42] Miller W, Buelow JM (2011). Psychometric testing of self-efficacy for partnership scale: an example of instrument development. Clinical Nurse Specialist.

[CR43] Moher D, Liberati A, Tetzlaff J, Altman DG, The PRISMA Group (2009). Preferred reporting items for systematic reviews and meta-analyses: The PRISMA Statement. PLoS Medicine.

[CR44] Moran TE, Polanin JR, Evenson AL, Troutman BR, Franklin CL (2016). Initial validation of the assessment of parenting tool: A task- and domain-level measure of parenting self-efficacy for parents of infants from birth to 24 months of age. Infant Mental Health Journal.

[CR45] Morawska A, Sanders MR, Haslam D, Filus A, Fletcher R (2014). Child adjustment and parent efficacy scale: Development and initial validation of a parent report measure. Australian Psychologist.

[CR46] Ohan JL, Leung DW, Johnston C (2000). The parenting sense of competence scale: Evidence of a stable factor structure and validity. Canadian Journal of Behavioural Science/Revue Canadienne des Sciences du Comportement.

[CR71] Pearsall, J., & Hanks, P. (1998). The New Oxford Dictionary of English. Oxford: Clarendon Press.

[CR47] Pennell C, Whittingham K, Boyd R, Sanders M, Colditz P (2012). Prematurity and parental self-efficacy: The preterm parenting & self-efficacy checklist. Infant Behavior and Development.

[CR48] Pridham KF, Chang AS (1989). What being the parent of a new baby is like: Revision of an instrument. Research in Nursing and Health.

[CR49] Purssell E, While A (2013). Parental self‐efficacy and its measurement–an evaluation of a parental self‐efficacy measurement scale. Journal of Clinical Nursing.

[CR50] Reece SM (1992). The parent expectations survey a measure of perceived self-efficacy. Clinical Nursing Research.

[CR51] Rogers, S. J., & White, L. K. (1998). Satisfaction with parenting: The role of marital happiness, family structure, and parents’ gender. *Journal of Marriage and the Family*, 293–308. http://www.jstor.org/stable/353849.

[CR52] Rutledge DL, Pridham KF (1987). Postpartum mothers’ perceptions of competence for infant care. Journal of Obstetric, Gynecologic, & Neonatal Nursing.

[CR53] Sanders MR (2000). Community-based parenting and family support interventions and the prevention of drug abuse. Addictive Behaviors.

[CR54] Sanders MR (2008). Triple p-positive parenting program as a public health approach to strengthening parenting. Journal of Family Psychology.

[CR55] Sanders MR, Woolley M (2005). The relationship between maternal self‐efficacy and parenting practices: Implications for parent training. Child: Care, Health and Development.

[CR56] Scientific Advisory Committee of the Medical Outcomes Trust (2002). Assessing health status and quality-of-life instruments: Attributes and review criteria. Quality of Life Research.

[CR57] Seashore MJ, Leifer AD, Barnett CR, Leiderman PH (1973). The effects of denial of early mother-infant interaction on maternal self-confidence. Journal of Personality and Social Psychology.

[CR58] Secco L (2002). The infant care questionnaire: Assessment of reliability and validity in a sample of healthy mothers. Journal of Nursing Measurement.

[CR59] Sevigny PR, Loutzenhiser L, McAuslan P (2016). Development and validation of the fathering self-efficacy scale. Psychology of Men & Masculinity.

[CR60] Swick, K. J., & Broadway, F. (1997). Parental efficacy and successful parent involvement. *Journal of Instructional Psychology*, *24*(1), 69. http://psycnet.apa.org/psycinfo/1997-07792-008.

[CR61] Terwee CB, Bot SD, de Boer MR, van der Windt DA, Knol DL, Dekker J, Bouter LM, de Vet HC (2007). Quality criteria were proposed for measurement properties of health status questionnaires. Journal of Clinical Epidemiology.

[CR62] Teti DM, Gelfand DM (1991). Behavioral competence among mothers of infants in the first year: the mediational role of maternal self‐efficacy. Child Development.

[CR72] Tucker, S., Gross, D., Fogg, L., Delaney, K., & Lapporte, R. (1998). The long-term efficacy of a behavioural parent training intervention for families with 2-year-olds. *Research in Nursing & Health*, *21*(3), 199–210. doi:10.1002/(SICI)1098-240X(199806)21:3<199::AID-NUR3>3.0.CO;2-C. 10.1002/(sici)1098-240x(199806)21:3<199::aid-nur3>3.0.co;2-c 9609505

[CR63] van Rijen E, Gasanova N, Boonstra A, Huijding J (2014). Psychometric qualities of the short form of the self-efficacy for parenting tasks index-toddler scale. Child Psychiatry & Human Development.

[CR64] Walker LO, Crain H, Thompson E (1986). Mothering behavior and maternal role attainment during the postpartum period. Nursing Research.

[CR65] Wittkowski A, Dowling H, Smith D, Wittkowski A (2016). Does engaging in a group-based intervention increase parental self-efficacy in parents of preschool children? A systematic review of the current literature. Journal of Child and Family Studies.

[CR66] Zahr LK (1991). The relationship between maternal confidence and mother–infant behaviors in premature infants. Research in Nursing and Health.

