# Supplementary material for: Self-Report Measures of Parental Self-Efficacy: A Systematic Review of the Current Literature
Source: J Child Fam Stud. 2017 Jul 6;26(11):2960–78. doi: 10.1007/s10826-017-0830-5 (PMC5646137; doi:10.1007/s10826-017-0830-5)
Supplement: Supplementary file 1 — Supplementary Table S1 [file 10826_2017_830_MOESM1_ESM.docx]

# Supplementary Table S1: Content and construct validity properties of the PSE measures

| Measure | Content validity | | | |  | Construct Validity | | | | | | | |
| --- | --- | --- | --- | --- | --- | --- | --- | --- | --- | --- | --- | --- | --- |
|  | Item development | Item reduction | Floor / Ceiling Effects* | Level of reading examined |  | Hypotheses | Convergent Validity | Discriminant Validity | Divergent Validity | Concurrent Validity | | | Predictive Validity |
|  |  |  |  |  |  |  |  |  |  | Sample | Comparative Measure(s) | Corr-elation |  |
| BaM-13 | Literature, author’s experience, discussed with team | ✓ | 0 | 0 |  | ✓ | ✓ | ✓ | 0 | 75 mothers | EPDS  KPCS | .64  .69 | 0 |
| BAP | 0 | 0 | 0 | 0 |  | 0 | 0 | 0 | 0 | 0 | 0 | 0 | 0 |
| CAPES | Expert consultation, parent consultation, investigators | ✓ | 0 | Age 13-15+ |  | 0 | ✓ | ✓ | 0 | 0 | 0 | 0 | 0 |
| C-G PSS | Focus groups, literature, pilot testing, expert consultation | ✓ | 0 | 0 |  | ✓ | 0 | 0 | 0 | 35 parents | DAS, MSM, LSI | .51, .46, .52 (p<.01) | 0 |
| CPP | Literature, expert consultation, parents | ✓ | 0 | 0 |  | 0 | 0 | 0 | 0 | 0 | 0 | 0 | 0 |
| EIPSES | Authors’ experience | ✓ | 0 | 0 |  | ✓ | 0 | 0 | 0 | Full sample | BDI,  ITSEA | 6% shared variance, r^2^ = .04 - .09 | 0 |
| ICQ | Author’s experience, literature | ✓ | 0 | 0 |  | ✓ | ✓ | 0 | 0 | Full sample | MCS | .48 (p=.000) | 0 |
| ICS | Expert consultation | ✓ | 0 | 0 |  | ✓ | ✓ | ✓ | 0 | 0 | 0 | 0 | 0 |
| KPCS | Focus group, expert consultation | ✓ | ✓ | 0 |  | ✓ | ✓ | ✓ | 0 | Full sample | PSOC, MEQ, PSI-SF, EPDS | .55, .62, -.63, -.56 (p<.01) | 0 |
| KPSS | Authors’ experience | ✓ | Ceiling | 0 |  | 0 | 0 | 0 | 0 | Full sample | SES 1980, 1984 | .23 (p=.02 to .42), .27 (p = .01 to .51) | 0 |
| MaMS & MBS | Authors’ experience, pilot testing | ✓ | 0 | 0 |  | 0 | 0 | 0 | 0 | Full sample | PSCS | 0 | 0 |
| MaaP | Literature, pilot testing, discussed with team, expert consultation, parent consultation | ✓ | 0 | 0 |  | ✓ | ✓ | 0 | 0 | Full sample | PSOC | .63 (p<.001) | 0 |
| MCQ | 0 | 0 | 0 | 0 |  | 0 | 0 | 0 | 0 | Full sample | NET-HELP  PSOC | .68 (p<.05)  .53 (p<.05) | 0 |
| MSEQ | 0 | 0 | 0 | 0 |  | 0 | 0 | 0 | 0 | 0 | 0 | 0 | 0 |
| M/P SES | 0 | 0 | 0 | 0 |  | 0 | 0 | 0 | 0 | Full sample | PSI** | -.75 (p<.001) | 0 |
| MIPSI | 0 | 0 | 0 | 0 |  | 0 | 0 | 0 | 0 | 0 | 0 | 0 | 0 |
| MSPC | 0 | 0 | 0 | 0 |  | 0 | 0 | 0 | 0 | 0 | 0 | 0 | ✓ |
| PCS | Literature, discussed with team, pilot testing | ✓ | 0 | 0 |  | 0 | ✓ | 0 | 0 | 0 | 0 | 0 | 0 |
| PEEM | Literature, discussed with team | ✓ | ✓ | 0 |  | ✓ | ✓ | 0 | 0 | 341, 435, 192 parents | FES, PSOC, WEMWBS | .66, .61, .59 (p<0.001) | 0 |
| PES | Literature, discussed with team, authors’ experience, expert consultation | ✓ | 0 | 0 |  | 0 | ✓ | ✓ | 0 | Full sample | WPBL-R | .57 | ✓ |
| PMP S-E | Literature, authors’ experience, Pilot testing | ✓ | ✓ | 0 |  | 0 | 0 | 0 | ✓ (n=60) | 60 mothers | MSRI, MPAS | .4 (p<.05), .31 (p<.01) | 0 |
| PPSEC | Literature, expert consultation | 0 | 0 | 0 |  | ✓ | 0 | 0 | 0 | Full sample | MSEQ | -.27 (p<.001) | 0 |
| PSAM | Literature, expert consultation | ✓ | 0 | 0 |  | ✓ | 0 | 0 | 0 | 0 | 0 | 0 | 0 |
| PSES | Expert consultation | 0 | ✓ | 0 |  | 0 | 0 | 0 | 0 | 0 | 0 | 0 | 0 |
| PSOC | 0 | 0 | 0 | 0 |  | ✓ | ✓ | 0 | ✓ | Full sample | PSOC (1989), CBCL, CRPR, DAS | p<.01 | 0 |
| PTC | Literature, discussed with team | 0 | 0 | 0 |  | 0 | ✓ | ✓ | 0 | 0 | 0 | 0 | 0 |
| SEPTI | 0 | 0 | ✓ | 0 |  | ✓ | 0 | 0 | 0 | 0 | 0 | 0 | 0 |
| SEPTI - TS | Literature | 🗶 | ✓ | 0 |  | 0 | ✓ | ✓ | 🗶 | 68 mothers | MEQ, SELF-ES  MSES, PSI** | .22 (p=.028), .32 (p=.009)  .60 (p<.001), -.72 (p<.001) | 0 |
| SICS | Literature | 0 | ✓ | 0 |  | ✓ | 0 | 0 | 0 | 135 mothers | PSI-SF | .92 | 0 |
| TCQ | Convenience Literature, Author’s experience, pilot testing, expert consultation | ✓ | 0 | 0 |  | 0 | ✓ | 0 | 0 | 0 | 0 | 0 | 0 |
| TOPSE | Focus groups, expert consultation, pilot testing | ✓ | 0 | 0 |  | 0 | 0 | ✓ | 0 | 0 | 0 | 0 | 0 |
| WPBL(R) | Pilot testing | ✓ | ✓ | 0 |  | ✓ | ✓ | 0 | 0 | 135 mothers | PPS | -.36 (p<.001) | 0 |

*Note.* BDI = Battelle Developmental Inventory, CBCL = Child Behavior Checklist, CRPR = Child Reading Practices Report; DAS = Dyadic Adjustment Scale; EPDS = Edinburgh Postnatal depression Scale; ITSEA = Infant-Toddler Social and Emotional Assessment; FES = Family Empowerment Scale); LSI = Life Satisfaction Index; MSM = Marital Satisfaction Measure; MSRI = Maternal Self-Report Inventory, MPAS = Maternal Postnatal Attachment Scale; NET-HELP = measure of social support for women and infants; PPS = How Parents Problem-solve Regarding the Infant; PSCS = Pharis Self Confidence Scale; PSI(SF) = Parenting Stress Index (Short Form); PSQ = Postpartum Self-Evaluation Questionnaire; PSS = Perceived Stress Scale; SES = Rosenberg Self Esteem Scale; SELF-ES = Self-Efficacy Scale; WEMWBS = Warwick-Edinburgh Mental Wellbeing Scale.

* ✓ = no floor or ceiling effect

** Competence scale
