# Supplementary material for: Self-Report Measures of Parental Self-Efficacy: A Systematic Review of the Current Literature
Source: J Child Fam Stud. 2017 Jul 6;26(11):2960–78. doi: 10.1007/s10826-017-0830-5 (PMC5646137; doi:10.1007/s10826-017-0830-5)
Supplement: Supplementary file 2 — Supplementary Table S2 [file 10826_2017_830_MOESM2_ESM.docx]

# Supplementary Table S2: Responsiveness and interpretability properties of the PSE measures

| Measure | Responsiveness and Interpretability | | | | | | | | | |
| --- | --- | --- | --- | --- | --- | --- | --- | --- | --- | --- |
|  | Cut off Score | Details | Area Under Curve | MCID | | | Responses | | | |
|  |  |  |  | 95% CI | 90% CI | Time Interval | M | SD | Comparative Data | Distribution of Scores |
| BaM-13 | 9+ | Sensitivity = 72.9%, Specificity: 74.4%, PPV: 34.9%, NPV: 93.5%, Misclassification Rate: 25.9%  Positive Likelihood Ratio: 2.83 | 0.81 (p<.001) | 4 points | 3 points | 2-4 weeks | ✓ | ✓ | 0 | 0 |
| BAP | 0 | 0 | 0 | 0 | 0 | 0 | 0 | 0 | 0 | 0 |
| CAPES | 0 | 0 | 0 | 0 | 0 | 0 | ✓ | ✓ | 0 | ✓ |
| C-G PSS | 0 | 0 | 0 | 0 | 0 | 0 | 0 | 0 | 0 | 0 |
| CPP | 0 | 0 | 0 | 0 | 0 | 0 | ✓ | 0 | 0 | 0 |
| EIPSES | 0 | 0 | 0 | 0 | 0 | 0 | ✓ | ✓ | ✓ | ✓ |
| ICQ | 0 | 0 | 0 | 0 | 0 | 0 | ✓ | 0 | ✓ | 0 |
| ICS | 0 | 0 | 0 | 0 | 0 | 0 | ✓ | 0 | 0 | 0 |
| KPCS | <39 | Sensitivity = 86%, Specificity = 89, PPV = 88%, NPV = 88%, Misclassification Rate: 12% | 0 | 6 points | 0 | 5 days | ✓ | ✓ | ✓ | 0 |
| KPSS | 0 | 0 | 0 | 0 | 0 | 0 | ✓ | ✓ | ✓ | 0 |
| MaMS &MBS | 0 | 0 | 0 | 0 | 0 | 0 | ✓ | ✓ | 0 | 0 |
| MaaP | 0 | 0 | 0 | 0 | 0 | 0 | ✓ | ✓ | 0 | 0 |
| MCQ | 0 | 0 | 0 | 0 | 0 | 0 | 0 | 0 | 0 | 0 |
| MSEQ | 0 | 0 | 0 | 0 | 0 | 0 | 0 | 0 | 0 | 0 |
| M/P SES | 0 | 0 | 0 | 0 | 0 | 0 | 0 | 0 | 0 | 0 |
| MIPSI | 0 | 0 | 0 | 0 | 0 | 0 | ✓ | ✓ | ✓ | 0 |
| MSPC | 0 | 0 | 0 | 0 | 0 | 0 | ✓ | ✓ | ✓ | 0 |
| PCS | 0 | 0 | 0 | 0 | 0 | 0 | ✓ | ✓ | ✓ | 0 |
| PEEM | 0 | 0 | 0 | 0 | 0 | 0 | ✓ | ✓ | 0 | ✓ |
| PES | 0 | 0 | 0 | 0 | 0 | 0 | 0 | 0 | 0 | 0 |
| PMP S-E | 0 | 0 | 0 | 0 | 0 | 0 | ✓ | ✓ | ✓ | ✓ |
| PPSEC | 0 | 0 | 0 | 0 | 0 | 0 | ✓ | ✓ | ✓ | 0 |
| PSAM | 0 | 0 | 0 | 0 | 0 | 0 | ✓ | ✓ | ✓ | ✓ |
| PSES | 0 | 0 | 0 | 0 | 0 | 0 | 0 | 0 | 0 | 0 |
| PSOC | 0 | 0 | 0 | 0 | 0 | 0 | ✓ | ✓ | ✓ | 0 |
| PTC | 0 | 0 | 0 | 0 | 0 | 0 | ✓ | ✓ | ✓ | 0 |
| SEPTI | 0 | 0 | 0 | 0 | 0 | 0 | ✓ | ✓ | ✓ | ✓ |
| SEPTI - TS | 118 | Normal sample: ≤10.5%  Clinical sample: ≤40.7% (P<.001) | 0 | 0 | 0 | 0 | ✓ | ✓ | ✓ | ✓ |
| SICS | 0 | 0 | 0 | 0 | 0 | 0 | ✓ | ✓ | ✓ | 0 |
| TCQ | 0 | 0 | 0 | 0 | 0 | 0 | 0 | 0 | 0 | 0 |
| TOPSE | 0 | 0 | 0 | 0 | 0 | 0 | ✓ | ✓ | ✓ | 0 |
| WPBL(R) | 0 | 0 | 0 | 0 | 0 | 0 | ✓ | ✓ | ✓ | 0 |

*Note.* Sensitivity = percentage of true cases correctly identified, Specificity = percentage of non-cases correctly identified, PPV = Positive Predictive Value (percentage of the sample scoring above the cut-off who were true cases), NPV = Negative Predictive Value (the percentage of the sample scoring below the cut-off who were true non-cases). CI = Confidence Intervals; M = Mean; SD = Standard Deviation
