# Supplementary material for: Self-Report Measures of Parental Self-Efficacy: A Systematic Review of the Current Literature
Source: J Child Fam Stud. 2017 Jul 6;26(11):2960–78. doi: 10.1007/s10826-017-0830-5 (PMC5646137; doi:10.1007/s10826-017-0830-5)
Supplement: Supplementary file 3 — Supplementary Table S3 [file 10826_2017_830_MOESM3_ESM.docx]

# Supplementary Table S3: Agreement and reliability properties of the PSE measures

| Measure | Agreement and Reliability | | | | | | | | | |
| --- | --- | --- | --- | --- | --- | --- | --- | --- | --- | --- |
|  | Test-Retest | | |  | Internal Consistency  Cronbach’s alpha | |  | Inter-item correlation matrix |  | Split Half |
|  | Sample | Correlation | Time Interval |  | Sample | α |  |  |  |  |
| BaM-13 | 47 mothers. .52 | .52 | 6 weeks |  | Full sample | .798 |  | No item redundancy. No two items correlating ≥.55. 73 of 78 correlations ≤.5 |  | 0 |
| BAP | 0 | 0 | 0 |  | 0 | 0 |  | 0 |  | 0 |
| CAPES | 0 | 0 | 0 |  | Full sample CAPES  Full sample Efficacy | .90  .96 |  | 0 |  | 0 |
| C-G PSS | 0 | 0 | 0 |  | Full sample | .927 |  | Spouse support and discipline =.66 (p<0.1), Parent-child relationship and satisfaction =.65 (p<.01) |  | 0 |
| CPP | 35 mothers | .85 (p=.001) | 2 weeks |  | 0 | 0 |  | 0 |  | 0 |
| EIPSES | 0 | 0 | 0 |  | Full sample | .81 |  | 0 |  | 0 |
| ICQ | 164 mothers | p≤.004 | 3-5 weeks |  | 164 mothers | .71. Scales: .86, .79, .58 |  | Correlation with time periods |  | 0 |
| ICS | 0 | 0 | 0 |  | Full sample | .975 |  | 0 |  | 0 |
| KPCS | 27 mothers | .88 (p<.001) | 4 weeks |  | Full sample | .81 (Scales: .44 to .80) |  | 0 |  | 0 |
| KPSS | 0 | 0 | 0 |  | 1980  1984 | .84  .84 (fathers), .78 (mothers) |  | 0 |  | 0 |
| MaMS & MBS | Full sample | None | 4-6 weeks |  | 0 | 0 |  | 0 |  | 0 |
| MaaP | 126 parents | .71 (p<.001) | 2-4 months |  | Full sample | .94 (Scales: .72 to .85) |  | 0 |  | 0 |
| MCQ | Full sample | .69 | 4 and 8 months |  | Full sample | .89 |  | Not significant |  | 0 |
| MSEQ | 0 | 0 | 0 |  | Full sample | .76, .73, .70 |  | 0 |  | 0 |
| M/P SES | 0 | 0 | 0 |  | 29 mothers, Full sample | .79, .86 |  | 0 |  | 0 |
| MIPSI | 0 | 0 | 0 |  | Full sample | .90 |  | 0 |  | 0 |
| MSPC | 0 | 0 | 0 |  | 0 | 0 |  | 0 |  | 0 |
| PCS | 0 | 0 | 0 |  | 58 mothers | .91 to .99 |  | 0 |  | 0 |
| PEEM | 200 parents | .84 (p<.001) | 4 weeks |  | Full sample | .92 |  | 0 |  | 0 |
| PES | 0 | 0 | 0 |  | Full sample | .91 (1 month), .86 (3 months) |  | 0 |  | 0 |
| PMP S-E | 100 mothers | .96 (p<.01) | 10 days |  | Full sample | .91 |  | .3-.77 (p=?) |  | 0 |
| PPSEC | 0 | 0 | 0 |  | Full sample | .75 |  | 0 |  | .75 |
| PSAM | 0 | 0 | 0 |  | Mexican Immigrant, English | .68, .70 |  | .43 to .56, .53 to .55 (p=?) |  | 0 |
| PSES | 0 | 0 | 0 |  | 0 | 0 |  | 0 |  | 0 |
| PSOC | 0 | 0 | 0 |  | Full sample | .77 to .80 |  | 0 |  | 0 |
| PTC | 0 | 0 | 0 |  | Full sample | Behavioural subscale: .97, Setting subscale: .91 |  | 0 |  | 0 |
| SEPTI | 0 | 0 | 0 |  | Full sample | .91 |  | Subscales: .27 to.55 (p<.01) |  | 0 |
| SEPTI - TS | 0 | 0 | 0 |  | Full sample | .91 (Subscales: .46 to .92) |  | Subscales: .26 to .71 (M=.43) |  | 0 |
| SICS | 23 mothers | .93 (p<.001) | 2 weeks |  | Full sample | .96 (Subscales: .86 to .93) |  | .41 Subscales: .44 to .56 |  |  |
| TCQ | Full sample | .87 | 4 weeks |  | Full sample | .95 |  | 0 |  | 0 |
| TOPSE | 19 mothers | Subscales: .58 to .88 (p<.01) | 4-6 weeks |  | Full sample | .95 |  | 0 |  | 0 |
| WPBL(R) |  |  |  |  | Full sample | .80 |  | .46 to .78 |  | 0 |

*Note.* A score of ‘0’ indicates that the information is missing
