# Supplementary material for: Self-Report Measures of Parental Self-Efficacy: A Systematic Review of the Current Literature
Source: J Child Fam Stud. 2017 Jul 6;26(11):2960–78. doi: 10.1007/s10826-017-0830-5 (PMC5646137; doi:10.1007/s10826-017-0830-5)
Supplement: Supplementary file 4 — Supplementary Table S4 [file 10826_2017_830_MOESM4_ESM.docx]

# Supplementary Table S4: Descriptive properties of the PSE measures

| Measure | | Sample | | | | | | | | |
| --- | --- | --- | --- | --- | --- | --- | --- | --- | --- | --- |
|  | Sample | Parent Age Range (years) | Child Age Range | Location | Language | Recruitment Location | Ethnicity, Other Demographics | Marital | Highest Level of Parent Education & Employment | Inclusion Criteria |
| BaM-13 | Mothers (n=630) | 0 | **0** | S.W Sydney (Australia) | English | Early childhood clinics (n=496), day-care (n=117), mental health services (n=17) | 0 | 0 | 0 | 0 |
| BAP | 0 |  |  | Australia | English |  |  |  |  |  |
| CAPES | Parents of 2-12-year-olds (n=347)  Mothers n=295), Fathers (n=14) | 24-58 (M=39.49, SD=5.98) | 2-12 (n=7.34, SD=2.80) | Australia | English | Schools, day care centres, online forum, parenting newsletters | Caucasian (n=250), Aboriginal (n=7), Asian (n=5), Other (n=8) | Married (n=229) | University Degree (n=166), High school (n=76), College or trade (n=67), In employment (n=239) | 0 |
| C-G PSS | Fathers (n=52), mothers (n=78) | 21-71 (M=38.76, SD=10.57) | 6 weeks to 38 years | USA | English | Educational, religious, community groups | Caucasian (90.8%), Protestant (58.5%), natural parents (93.8%) | Children in family 1-11 (m=3, SD=5.31).  Married (94%), employed (83%), | Years of education m=15.92, SD=3.55) | 0 |
| CPP | Mothers (n=278) | 0 | 0 | 0 | English | Private paediatric clinic | Caucasian (90%+) | Married (90%+) | High school (90%+) | Child ≤ 18years, no illness or disability |
| EIPSES | Biological mothers (n=112), adoptive mothers (n=5) | 16-52 (M = 31.11, SD = 6.99) | 3months – 34 months (n=17.28, SD = 6.82) | Arizona, Utah, N.California (USA) | English, Spanish | Local agencies | White (65.8%), Hispanic / Latino (23.1%), other (11.1%) | Two-parent household (88.9%) | Higher degree (33%), College (27%), College degree (9.6%),  High school (23.5%), < high school (5.2%) | Child has a disability* |
| ICQ | Mothers (n=264) | 28 | 0 | Manitoba, Canada | English | Postpartum unites at two teaching hospitals | Multiparous (n=48), Primparous (n=114), Caucasian (95%) | Married (88%) | ≥ High school | Infants of at least 37 weeks, no illness or disability. |
| ICS | Mothers and Fathers (m=142) | 15-40+ | 0 | 0 | 0 | Hospitals, homes, classrooms | Caucasian, Black, Hispanic | 0 | Middle school to College Graduate | 0 |
| KPCS | mothers (n=187) | 18+ (M=32, SD=4.8) | 24.7 weeks (SD=25.5) | Sydney, Australia | English | Community and Karitane, Caring for Families | Australian (63%) | Married (94%), Number of children M=1.5 (SD=.8) | Completed university or vocational course (92%) | 0 |
| KPSS (1980) | 84 mothers | 0 | 0 | Kansas | English | Random | Caucasian, Protestant, middle-class | 0 | 0 | 0 |
| (1984) | 52 fathers, 85 mothers | 20-29 (n=24.2%), 30-39 (50.4$, 40-49 (26.05%), 50-59 (3.95%) | 0 | Southern Baptist Church | English | Parents’ enrichment programme sponsored by Southern Baptist Convention | 0 | 1.76 children living at home (SD = .89) | Higher degree (27.2%), College (30.6%), College degree (21.8%), < high school (17.8%), In employment (66.5%) | 0 |
| MaMS & MBS | 122 mothers | Primiparas: 17-41 (M=25.7)< multiparas 19-40 (M=29.2) | 0 | 0 | English | Community hospital | White | Married. First time mothers (n=64) | College education | Medically uneventful pregnancy, full-term singletons, no illnesses or disability |
| MaaP | 300 parents | 21-74 (M=40.95, SD=7.50) | 6 months – 15 years | Australia | English | Over telephone via Random Digit Dialling | Child with a disability (12%) | Married (77%) | Bachelor’s degree (42%), High School (47.3%). In full-time employment (38%), in part-time employment (28%) | Parent aged at least 18 years, with child aged 6 months to 15 years. |
| MCQ | 43 mothers | 18-47 (M=24.25, SD=5.8) | Gestational age (m=30.32), birth weight (M=1.50kg) | 0 | English | Inner city hospital | 0 | 0 | M=10.5 years of education | Birth weight ≤2kg, medically uneventful, no mother or child illness or disability |
| MSEQ | 83 mothers | 16-42 (M=28.96, SD=4.60) | Gestational age 36-43 weeks (M=39.78 weeks, SD 1.52) | 0 | English | 0 | Caucasian (98%) | Married (87%) | M=15.06 years of education (SD=2.89) | 0 |
| M/P SES | 86 mothers (48 diagnosed with depression) | 16-17 (n=3), 18-40 (n=83) | 3-13 months (M=7.35 months) | 0 | English | 0 | White (97.35%), Black (2.6%), Hispanic (2.6%). Religion: Mormon (61.9%), Catholic (10.2%), Protestant (8.4%), Other (19.7%) | Single (16%), Married/Living with partner (84%) | Graduate/Professional (3.55%), College graduate (24.3%), College / vocational (45.3%), High school (13.0%), < High school (7.2%) | 0 |
| MIPSI | 161 two parent families (n=322) | 0 | 0 | SW USA | English, Spanish | Low income, inner city school rosters | European American Parents (n = 93), Mexican American parents (English) (n = 68), Mexican American parents (Spanish) (n=161) | 0 | European American (M=12.85 years of education), Mexican American (English) M=11.8, Mexican American (Spanish) M = 7.25 years) | 0 |
| MSPC | 43 mothers (21 denied physical contact with child) | 0 | Two weeks premature to 1 month | 0 | English | Hospital | 0 | 0 | 0 | No previous history of premature births, newborn weighs 890-1,899g, no illness or disability, father present |
| PCS | 140 mothers | M=28.3 (SD = 4.62) | Premature (<38 weeks, small (term, <5lb) or in intensive care (n=41) | Midwest USA | English | Postpartum unit of community hospital affiliated with university | Caucasian (94%). Religion: Protestant (39%), Catholic (35%), Other (26%). | Married (93%) | M=14.5 years of education (SD=2.57) | 0 |
| PEEM | 866 parents and carers |  | 5-12 years | Australia | English | Participating schools | High (n=290), medium (n=228), low (n=348) SES | Single parent (17.7%), grandparent / guardian (2%), 1.5 children living at home (SD=.70) | University qualification (35.1%), < high school (11.6%), trade (53.2%) | 0 |
| PES | 105 mothers | 35 to 42 (M=37) | 0-1 year | Boston, USA | English | Childbirth education programmes in hospitals | 0 | 0 | College educated (“most”) | First time parent |
| PMP S-E | 160 mothers | 0 | 0-28 days postnatal | UK | English | 0 | White (86%) | First time mother (56%) | Worked during pregnancy (44%), smoked during pregnancy (31%), worked and smoked during pregnancy (14%) | English, no illness or disability, preterm baby (<2.5kg, <37 weeks gestational) |
| PPSEC | 152 mothers, 3 fathers | M= 31.6, (SD =5.27) | 14.08 months (SD (6.72) |  |  | Neonatal Unit, Royal Brisbane & women’s Hospital |  | First time parent (n=78), single birth (n=134) |  |  |
| PSAM | 94 Spanish (Mexican Immigrant) & 90 English speaking families | Median = 31-35 & 36-40 | 9-10, 3-12 | USA | Spanish & English | 0 | 0 | Married (68% & 82%) | Median = < 15 years & bachelor’s degree | 0 |
| PSES | 152 parents |  | 0-6 years | London, UK | English | Parenting & community organisations, online | White (83%), Black African (11%), Black Caribbean (3%) | 2 (61%), 3 (22%), 4 (12%) children | A-Level (24%), Graduate (56%), Post-graduate (20%) | Child experiences periods of minor illness** |
| PSOC | 110 couples | 26-49 (M=36-6, SD=3.95) | 5-12 years | Large urban | English | Posters in community centres and newspapers | 0 | 1.4 additional siblings at home (SD = .76) | 0 | 0 |
| PTC | 124 mothers (79 community sample, 45 clinic sample) | M=34.31 (SD=5.66) | 2-8 years (M=4.19, SD=3.06) | Brisbane, Australia | English | Community: child care centres, kindergartens  Clinic: Triple P Positive Parenting Program at Parenting and Family Support Centre, University of Queensland | 0 | Single parent (n=22), Married (n=102)  1.16 siblings (SD=.33) | University degree (n=52), Trade (n=3), college (n=27), year 12 (24), <year 11 (n=17) | 0 |
| SEPTI | 145 mothers |  | 5-12 years | West Virginia | English | Public elementary school | White (95%) | Married (72%), | Unemployed (35%, part-time (215), full time (43%) | 0 |
| SEPTI - TS | 309 parents (282 normal sample, 27 clinical sample) | M=33.8, SD=5.1 | 17-48 months | Rotterdam & Barendrecht (S Holland) | Dutch | Day care centres (normal sample), day treatment program for children with psychiatric problems (clinical sample) |  | Additional siblings 0 (n=103), 1 (n=162), 2 (n=32), 3+ (n=4) | Educational attainment = Low (25.3%), Middle (37.2%), High (37.5%). Employed (86.4%) | 0 |
| SICS | 235 mothers | 15-44 (M=25.4, SD=5.98) | M=226.24 days (SD=60.38 days) | Samutsakorn Province, Bangkok, Thailand | Thai | Samutsakorn Hospital | 0 | First time mothers (65.5%) | Education (M=10.01, SD=3.42). Employed (61.7%) | Mothers had to read and write, no illness or disability, |
| TCQ | 49 mothers | 25-48 (M=33) | M=23 months | 0 | English | 0 | 0 | Married (100%) (M=8 years). 1^st^ born (n=32), 2^nd^born (n=17) | Graduate (36.7%), College (30.6%, Some college or high school (32.7%).  Unemployed = 58% | One child between 12 and 36 months |
| TOPSE | 82 parents | 22-52 (M=35) | 0 | UK | English | Heath visitor caseloads in 3 primary care trusts | White (n=56) | Female (n=58), married or living with partner (n=56). Children in family = 1to5 (m=2) | Education > 16 years (n=29). Full or part-time employment (n=27) | Child up to 6 years |
| WPBL(R) | 93 mothers | 27.4 (SD=4.3) | 0 | Wisconsin, USA | English | 0 | 0 | First time mothers (n=46) | Education M =14.4 years | 0 |

*Note.* A score of ‘0’ indicates that the information is missing.

*Disabilities included Down syndrome (n=14), physical disability (n=23), epilepsy (n=8), vision impairment (n=18), cerebral palsy (n=11), speech/hearing impairment (n=11), chronic illness (n=4), mental retardation (n=9), developmental delay (n=75), other delay (n=24)

** Minor illnesses require no or very limited medical intervention
